# Effects of kangaroo mother care on maternal and paternal health: systematic review and meta-analysis

**DOI:** 10.2471/BLT.22.288977

**Published:** 2023-03-31

**Authors:** Barsha Gadapani Pathak, Bireshwar Sinha, Neeraj Sharma, Sarmila Mazumder, Nita Bhandari

**Affiliations:** a Centre for Health Research and Development, Society for Applied Studies, 45 Kalu Sarai, New Delhi, 110016, India.

## Abstract

**Objective:**

To investigate the effect of kangaroo mother care for low-birth-weight and preterm infants on parents’ mental and physical health.

**Methods:**

The Cochrane Central Register of Controlled Trials, Cochrane Register of Studies Online, PubMed^®^, Web of Science, Scopus and EMBASE^®^ databases were searched on 16 January 2023 for randomized and quasi-randomized trials on kangaroo mother care. Records identified were screened independently by two reviewers. Pooled relative risks (RRs) are reported for categorical variables, and standardized mean differences (SMDs) or mean differences are reported for continuous variables. Evidence quality was assessed using the GRADE approach.

**Findings:**

The search identified 30 studies involving 7719 preterm or low-birth-weight infants. There was high-certainty evidence that kangaroo mother care substantially reduced the risk of moderate-to-severe postpartum maternal depressive symptoms compared with no kangaroo mother care (RR: 0.76; 95% confidence interval, CI: 0.59 to 0.96). In addition, there was low-certainty evidence that kangaroo mother care reduced scores for maternal stress (SMD: −0.82; 95% CI: −1.32 to −0.32) and anxiety (SMD: −0.62; 95% CI: −1.01 to −0.23) and increased mother–infant attachment and bonding scores (SMD: 1.19; 95% CI: 0.27 to 2.10). Limited evidence indicated father–infant interactions may be improved, though no marked effect on paternal depression or stress was observed. No trial reported parental physical health outcomes.

**Conclusion:**

Kangaroo mother care for preterm and low-birth-weight infants was associated with less postpartum maternal depression, stress and anxiety and better mother–infant attachment and bonding. More research is required to evaluate effects on paternal health.

## Résumé

**Objectif:**

Étudier les effets, sur la santé mentale et physique des parents, de la méthode «mère kangourou» appliquée aux nouveau-nés prématurés ou de faible poids de naissance.

**Méthodes:**

Nous avons parcouru les bases de données du Registre central Cochrane des essais contrôlés, du Registre Cochrane des études en ligne, de PubMed^®^, de Web of Science, de Scopus et d'EMBASE^®^ le 16 janvier 2023, à la recherche d'essais randomisés et quasi-randomisés sur la méthode «mère kangourou». Les documents retenus ont été passés en revue séparément par deux réviseurs. Les risques relatifs combinés (RR) ont été signalés pour les variables nominales, tandis que les différences moyennes standardisées (DMS) ou différences moyennes l'ont été pour les variables continues. Enfin, la qualité des données probantes a été évaluée selon l'approche GRADE.

**Résultats:**

La recherche a permis d'identifier 30 études impliquant 7719 nouveau-nés prématurés ou de faible poids de naissance. Nous pouvons affirmer avec un degré élevé de certitude que le risque d'apparition des symptômes d'une dépression post-partum modérée à sévère est nettement plus faible chez les mères ayant adopté la méthode «mère kangourou» que chez celles ne l'ayant pas adoptée (RR: 0,76; intervalle de confiance de 95%, IC: 0,59 à 0,96). En outre, il existe des preuves de faible certitude selon lesquelles la méthode «mère kangourou» ferait baisser le niveau de stress maternel (DMS: -0,82; IC de 95%: −1,32 à −0,32) et d'anxiété (DMS: -0,62; IC de 95%: −1,01 à −0,23), tout en renforçant les relations mère-enfant et la formation de liens affectifs (DMS: 1,19; IC de 95%: 0,27 à 2,10). Des données limitées indiquent que les interactions père-enfant pourraient s'en trouver améliorées, même si aucun impact significatif n'a été constaté sur le stress ou la dépression paternelle. Aucun essai n'a fait état d'une influence sur la santé physique des parents.

**Conclusion:**

La méthode «mère kangourou» appliquée aux nouveau-nés prématurés ou de faible poids de naissance était associée à une diminution du stress, de l'anxiété et de la dépression post-partum du côté maternel, ainsi qu'à un attachement et des liens plus forts entre la mère et l'enfant. D'autres recherches sont nécessaires pour évaluer ses effets sur la santé paternelle.

## Resumen

**Objetivo:**

Investigar los efectos del método madre canguro para recién nacidos prematuros y de bajo peso al nacer sobre la salud mental y física de los padres.

**Métodos:**

Se realizaron búsquedas en las bases de datos del Registro Cochrane Central de Ensayos Controlados, del Registro Cochrane de Estudios en Línea, de PubMed®, de Web of Science, de Scopus y de EMBASE® el 16 de enero de 2023 para obtener ensayos aleatorizados y cuasialeatorizados sobre el método madre canguro. Dos revisores analizaron de forma independiente los registros identificados. Se informan los riesgos relativos (RR) agrupados para las variables categóricas y las diferencias de medias estandarizadas (DME) o las diferencias de medias para las variables continuas. La calidad de la evidencia se evaluó mediante el enfoque GRADE.

**Resultados:**

La búsqueda identificó 30 estudios con 7719 recién nacidos prematuros o de bajo peso al nacer. Hubo pruebas de alta certeza de que el método madre canguro redujo significativamente el riesgo de síntomas depresivos posparto de moderados a graves en comparación con ningún método madre canguro (RR: 0,76; intervalo de confianza del 95%, IC: 0,59 a 0,96). Además, hubo pruebas de baja certeza de que el método madre canguro redujo las puntuaciones de estrés materno (DME: -0,82; IC del 95%: -1,32 a -0,32) y ansiedad (DME: -0,62; IC del 95%: -1,01 a -0,23) y aumentó las puntuaciones de apego y vinculación madre-hijo (DME: 1,19; IC del 95%: 0,27 a 2,10). Las pruebas limitadas indicaron que las interacciones padre-hijo pueden mejorar, aunque no se observó un efecto marcado sobre la depresión o el estrés paterno. Ningún ensayo informó desenlaces de salud física de los padres.

**Conclusión:**

El método madre canguro para recién nacidos prematuros y de bajo peso al nacer se asoció con menos depresión, estrés y ansiedad maternos, posparto y mejor apego y vinculación madre-hijo. Se necesitan más investigaciones para evaluar los efectos sobre la salud paternal.

## ملخص

الغرض تقصي تأثير رعاية الأم لوليدها على طريقة الكنغر للأطفال ذوي الوزن المنخفض عند الولادة، والأطفال المبتسرين، على الصحة العقلية والبدنية للوالدين.

الطريقة تم البحث في قواعد بيانات كل من سجل كوكرين المركزي للتجارب ذات الشواهد، وسجل كوكرين للدراسات عبر الإنترنت، وPubMed ^® ^‎، وWeb of Science، وScopus، وEMBASE ^® ^‎، وذلك في 16 يناير/كانون ثاني 2023 للتجارب العشوائية وشبه العشوائية على رعاية الأم لوليدها على طريقة الكنغر. تم فحص السجلات المحددة بشكل مستقل بواسطة اثنين من المراجعين. تم الإبلاغ عن المخاطر النسبية المجمعة (RR) للمتغيرات الفئوية، والفروق المتوسطة المعيارية (SMD)، أو تم الإبلاغ عن الفروق المتوسطة للمتغيرات المستمرة. تم تقييم جودة الأدلة باستخدام نهج GRADE.

النتائج حدد البحث 30 دراسة شملت 7719 رضيعًا مبتسرًا أو منخفضي الوزن عند الولادة. كان هناك دليل بدرجة عالية من الثقة على أن رعاية الأم لوليدها على طريقة الكنغر، قللت بشكل ملموس من خطر الأعراض المعتدلة إلى الشديدة لاكتئاب ما بعد الولادة لدى الأم، مقارنة مع عدم وجود رعاية الأم لوليدها على طريقة الكنغر (نسبة المخاطر: 0.76؛ بفاصل ثقة مقداره %95: 0.59 إلى 0.96). بالإضافة إلى ذلك، كان هناك دليل بدرجة منخفضة من الثقة على أن رعاية الأم لوليدها على طريقة الكنغر قللت من درجات إجهاد الأم (فرق المتوسط المعياري: -0.82؛ ‏بفاصل ثقة مقداره %95: -1.32 إلى -0.32) والقلق (فرق المتوسط المعياري: -0.62؛ ‏بفاصل ثقة مقداره %95: -1.01 إلى -0.23) وزيادة درجة الارتباط والترابط بين الأم والرضيع (فرق المتوسط المعياري: 1.19؛ ‏بفاصل ثقة مقداره %95: 0.27 إلى 2.10). هناك أدلة محدودة تشير إلى أنه يمكن تحسين التفاعلات بين الأب والرضيع، على الرغم من عدم ملاحظة أي تأثير ملحوظ على اكتئاب أو إجهاد الأب. لم تسجل أية تجربة نتائج للصحة البدنية للوالدين.

الاستنتاج ارتبطت رعاية الأم لوليدها، المبتسر أو منخفض الوزن عند الولادة، على طريقة الكنغر بقدر أقل من اكتئاب وتوتر وقلق ما بعد الولادة لدى الأم، وارتباط وترابط أفضل بين الأم والرضيع. هناك حاجة للمزيد من الأبحاث بغرض تقييم الآثار على صحة الأب.

## 摘要

**目的:**

旨在探究对低出生体重儿和早产儿进行袋鼠式护理对父母身心健康的影响。

**方法:**

于 2023 年 1 月 16 日检索了 Cochrane 临床对照试验中心注册数据库、Cochrane 在线研究注册库、PubMed^®^、Web of Science、Scopus 和 EMBASE^®^ 数据库中有关袋鼠式护理的随机和半随机试验。由两名审查人员独立完成对已识别记录的筛选。分类变量报告合并相对危险度 (RR),连续变量报告标准化均数差 (SMD) 或均值差。使用 GRADE 方法评估证据质量。

**结果:**

通过检索确定了 30 项研究,涉及 7719 名早产儿或低出生体重儿。有高确定性证据表明,相比于未进行袋鼠式护理的情况,袋鼠式护理大大降低了母亲患有中度至重度产后抑郁症状的风险(RR:0.76;95% 置信区间,CI:0.59 至 0.96)。此外,有低确定性证据表明,袋鼠式护理降低了母亲的压力评分(SMD:-0.82;95% CI:-1.32 至 -0.32)和焦虑评分(SMD:-0.62;95% CI:-1.01 至 -0.23)并增加了母婴依恋和亲密关系评分(SMD:1.19;95% CI:0.27 至 2.10)。有限的证据表明,父亲与婴儿的互动可能会得到改善,但没有观察到对父亲的抑郁或压力有显著影响。没有试验报告父母身体健康的结果。

**结论:**

对早产儿和低出生体重儿进行袋鼠式护理与缓解母亲产后抑郁、压力和焦虑以及改善母婴依恋和亲密关系有关。需要更多的研究才能评估对父亲健康的影响。

## Резюме

**Цель:**

Изучить влияние метода «кенгуру» для ухода за детьми с низкой массой тела и недоношенными младенцами на психическое и физическое здоровье родителей.

**Методы:**

Поиск рандомизированных и квазирандомизированных исследований по методу «кенгуру» был проведен в базах данных Cochrane Central Register of Controlled Trials, Cochrane Register of Studies Online, PubMed®, Web of Science, Scopus и EMBASE® 16 января 2023 года. Отобранные записи были проверены независимо друг от друга двумя рецензентами. Объединенные относительные риски (ОР) представлены для категориальных переменных, а стандартизированные разности средних (ССР) или разности средних приведены для непрерывных переменных. Качество доказательств оценивали с использованием подхода GRADE.

**Результаты:**

В ходе поиска было отобрано 30 исследований с участием 7719 недоношенных или новорожденных с низкой массой тела. Были получены доказательства высокой степени достоверности того, что метод «кенгуру» существенно снижает риск развития послеродовой депрессии от умеренной до тяжелой степени у матерей по сравнению с уходом, в котором не использовался метод «кенгуру» (ОР: 0,76; 95%-й ДИ: от 0,59 до 0,96). Кроме того, были получены данные низкой степени достоверности о том, что применение метода «кенгуру» снижало показатели материнского стресса (SMD: −0,82; 95%-й ДИ: от −1,32 до −0,32) и тревожности (SMD: −0,62; 95%-й ДИ: от −1,01 до −0,23) и повышало показатели привязанности и связи между матерью и младенцем (SMD: 1,19; 95%-й ДИ: от 0,27 до 2,10). Ограниченные данные указывают на возможность улучшения взаимодействия между отцом и ребенком, хотя заметного влияния на депрессию или стресс отца не наблюдалось. Ни в одном исследовании не сообщалось о показателях физического здоровья родителей.

**Вывод:**

Использование метода «кенгуру» для ухода за недоношенными детьми и детьми с низкой массой тела при рождении сопровождалось меньшей послеродовой материнской депрессией, стрессом и беспокойством, а также улучшением степени привязанности и связи между матерью и ребенком. Для оценки влияния на здоровье отцов требуется проведение дополнительных исследований.

## Introduction

Pregnancy and childbirth are critical periods in women’s lives involving major physiological, psychological, domestic and sociodemographic changes. During the first 6 months postpartum,^1^ an estimated 15% to 33% of mothers experience anxiety and, during the first year after birth, around one fifth have postpartum depressive symptoms.^2^ The prevalence of depressive symptoms and anxiety seems to be even higher among mothers whose infants are born preterm (i.e. under 37 weeks’ gestation) or have a low birth weight (i.e. under 2500 g) compared to those whose infants are born at full term and have a normal birth weight.^3^^–^^5^ In addition, the birth of a preterm or low-birth-weight baby can also have consequences for the father’s mental health and have a negative impact on family life.^5^^,^^6^

Kangaroo mother care is an intervention that involves continuous skin-to-skin contact of the infant with the mother’s chest (or the chest of another caregiver when the mother is unavailable) and exclusive breastfeeding. The World Health Organization (WHO) recommends early and prolonged kangaroo mother care for low-birth-weight and preterm infants as it has been shown to reduce the risk of neonatal and infant death and to prevent infection.^7^^–^^9^ Although the beneficial effects of the intervention on infant health have been reviewed rigorously,^8^^,^^9^ its potential benefits for mothers and fathers are less well understood, which often presents a barrier to the promotion of kangaroo mother care.^10^ Previous reviews of the effect of kangaroo mother care on maternal health outcomes have either not involved a meta-analysis (i.e. no pooled estimates),^11^ been limited to only specific health outcomes (e.g. the mean maternal depression score),^12^ or not included all preterm and low-birth-weight infants.^13^ In addition, these reviews have not reported paternal health outcomes. There is a need, therefore, for a rigorous and updated evidence synthesis that comprehensively summarizes the full range of benefits provided by kangaroo mother care for both maternal and paternal health. This information will be important for updating recommendations for kangaroo mother care that reflect improvements in maternal health in addition to benefits for the child.

The primary aim of our study was to supplement existing knowledge on kangaroo mother care by performing a comprehensive and up-to-date literature review and meta-analysis of the impact of kangaroo mother care for low-birth-weight and preterm infants on the mothers’ mental and physical health. In addition, we investigated the effect of the practice on bonding between mother and infant and on paternal mental and physical health. We also conducted a quality assessment using the Grading of Recommendations Assessment, Development and Evaluation (GRADE) approach to evaluate the certainty of the pooled estimates,^14^ which has not been done in any prior systematic review.

## Methods

We searched the Cochrane Central Register of Controlled Trials (CENTRAL), Cochrane Register of Studies Online, PubMed^®^, Web of Science™, Scopus and EMBASE^®^ databases for articles on randomized controlled trials or quasi-randomized trials published before 16 January 2023 that compared kangaroo mother care with no kangaroo mother care for preterm or low-birth-weight infants. Details of the search strategies are provided in Box 1. There were no date or language restrictions in the search strategy. Articles written in a language other than English were reviewed and data were extracted from the English abstract where available. If an abstract was not in English, an online translation application was used. If it was still not possible to extract the relevant information, the article was excluded. In addition, the reference lists of the articles selected were searched manually to identify further relevant articles. This review was registered in the PROSPERO prospective register of systematic reviews (CRD42022323152) in accordance with the Preferred Reporting Items for Systematic Reviews and Meta-Analyses Protocol.^15^

Box 1 **Literature search strategy, meta-analysis of the maternal and paternal effects of kangaroo mother care for low-birth-weight and preterm infants, 1988–2023**

**PubMed®**

Search 1 (intervention; 3495 records identified)

Search terms: (“kangaroo mother care method”[MeSH Terms] OR “kangaroo-mother care method”[MeSH terms] OR “skin to skin contact”[Title/Abstract] OR “skin-to-skin contact”[Title/Abstract] OR “skin to skin care”[Title/Abstract] OR “skin-to-skin care”[Title/Abstract] OR “kangaroo mother care”[Title/Abstract] OR “kangaroo care”[Title/Abstract] OR “kangaroo”[Title/Abstract] OR “kangaroo holding”[Title/Abstract])

Search 2 (randomized and non-randomized clinical trials; 1 497 612 records identified)

Search terms: (Randomized Controlled Trial [Publication type] OR controlled clinical trial [Publication type] OR Clinical Trial [Publication type] OR randomized [Title/Abstract] OR placebo [Title/Abstract] OR clinical trials as topic [MeSH: noexp] OR randomly [Title/Abstract] OR trial [Title] OR Non-Randomized Controlled Trials as Topic”[MeSH]) NOT (animals [MeSH] NOT humans [MeSH])

Search 1 AND 2 (final search; 441 records identified)

**Cochrane Library (330 records identified)**

Search terms: For the Cochrane Central Register of Controlled Trials (CENTRAL) database, we used the pre-identified MeSH terms “kangaroo mother care method” and “kangaroo care” with no limitations on language or date of publication.

**Web of Science (342 records identified)**

Search terms: ((ALL = (“Randomized Controlled Trial” OR “controlled clinical trial” OR “Clinical Trial” OR randomized OR placebo OR “clinical trials as topic” OR randomly OR trial OR “Non-Randomized Controlled Trials as Topic”)) NOT ALL = ((animals NOT humans))) AND TI = ((“kangaroo mother care method” OR “kangaroo-mother care method” OR “skin to skin contact” OR “skin-to-skin contact” OR “skin to skin care” OR “skin-to-skin care” OR “kangaroo mother care” OR “kangaroo care” OR “kangaroo” OR “kangaroo holding”)) AND Articles (Document Types)

**EMBASE® (551 records identified)**

Search terms: ('kangaroo mother care method':ti,ab OR 'kangaroo-mother care method':ti,ab OR 'skin to skin contact':ti,ab OR 'skin-to-skin contact':ti,ab OR 'skin to skin care':ti,ab OR 'skin-to-skin care':ti,ab OR 'kangaroo mother care':ti,ab OR 'kangaroo care':ti,ab OR 'kangaroo':ti,ab OR 'kangaroo holding':ti,ab) AND ('randomized controlled trial':ti,ab OR 'controlled clinical trial':ti,ab OR 'clinical trial':ti,ab OR randomized:ti,ab OR placebo:ti,ab OR 'clinical trials as topic':ti,ab OR randomly:ti,ab OR trial:ti,ab OR 'non-randomized controlled trials as topic':ti,ab) NOT animals:ti,ab

**Scopus (552 records identified)**

Search terms: TITLE-ABSTRACT ((“kangaroo mother care method” OR “kangaroo-mother care method” OR “skin to skin contact” OR “skin-to-skin contact” OR “skin to skin care” OR “skin-to-skin care” OR “kangaroo mother care” OR “kangaroo care” OR “kangaroo” OR “kangaroo holding”) AND (“Randomized Controlled Trial” OR “controlled clinical trial” OR “Clinical Trial” OR randomized OR placebo OR “clinical trials as topic” OR randomly OR trial OR “Non-Randomized Controlled Trials as Topic”) NOT (animals))

MeSH: medical subject heading; ti,ab: title, abstract.Note: The search was performed on 16 January 2023.

We included studies that defined kangaroo mother care as skin-to-skin contact accompanied by the promotion of, or support for, exclusive breastfeeding. Kangaroo mother care could be initiated in either a hospital or a community setting, and could be initiated either immediately after birth or when low-birth-weight or preterm infants were in a stable condition. We excluded observational and crossover trials, and trials involving infants born at full term or with a normal birth weight.

The primary outcomes studied were maternal mental health outcomes, including moderate or severe postpartum depressive symptoms, and scores for postpartum depressive symptoms, stress, anxiety and distress. Secondary outcomes included: mother–infant attachment and bonding scores; paternal mental health outcomes; and maternal physical health outcomes such as breast problems (e.g. abscess or engorgement), postpartum bleeding and uterine involution. All outcomes were reported at the latest follow-up.

Data were reviewed using Covidence systematic review software (Veritas Health Innovation, Melbourne, Australia). Two authors independently screened titles and abstracts to identify relevant citations before carrying out full text reviews using predefined inclusion criteria. Data were extracted using a modified version of the Cochrane Effective Practice and Organization of Care (EPOC) group data collection checklist (Cochrane EPOC Group, London, United Kingdom of Great Britain and Northern Ireland),^16^ and included study identifiers and context, study design, intervention details, outcome assessment tools and study outcomes. Any disagreements or discrepancies between reviewers were resolved by discussion or on review by a third author.

### Data analysis

For the data analysis, we followed the recommendations of the Cochrane Handbook for Systematic Reviews of Interventions.^17^ The analysis was performed using Stata version 16 (StataCorp LLC, College Station, United States of America). Pooled relative risks (RRs) are reported for categorical variables and mean differences for continuous variables, both with 95% confidence intervals (CIs). The standardized mean difference (SMD) served as a summary statistic when studies used different psychometric scales for assessing an outcome.^18^ The SMD was calculated as the mean difference between the intervention and control group means in each trial divided by their respective standard deviations.^18^ A fixed-effects meta-analysis (inverse variance method) was used to pool data and estimate effects. However, if the heterogeneity between studies was high (i.e. *I^2^* was greater than 50%),^19^ we used a random-effects model with the restricted maximum likelihood method. Egger’s test was used to assess publication bias for outcomes reported in at least five studies.

The risk of bias in included studies was assessed using the revised Cochrane risk-of-bias tool for randomized trials (RoB 2) or the risk-of-bias tool for nonrandomized studies of interventions (ROBINS-I), as appropriate (Cochrane, London, United Kingdom).^15^ The certainty of the evidence for the pooled estimates of outcomes was assessed using the GRADE approach.^14^

Prespecified subgroup analyses were performed for: (i) the location where kangaroo mother care was initiated (i.e. in hospital or the community); (ii) the provider of kangaroo mother care (i.e. the mother alone, the mother supported by the father or the mother supported by other caregivers); (iii) the time of outcome assessment (i.e. when the infant was younger than 6 months, 6 to 12 months or older than 12 months); (iv) country income level (i.e. high, middle or low income); and (v) the type of outcome assessment scale.

## Results

**Fig. 1:**
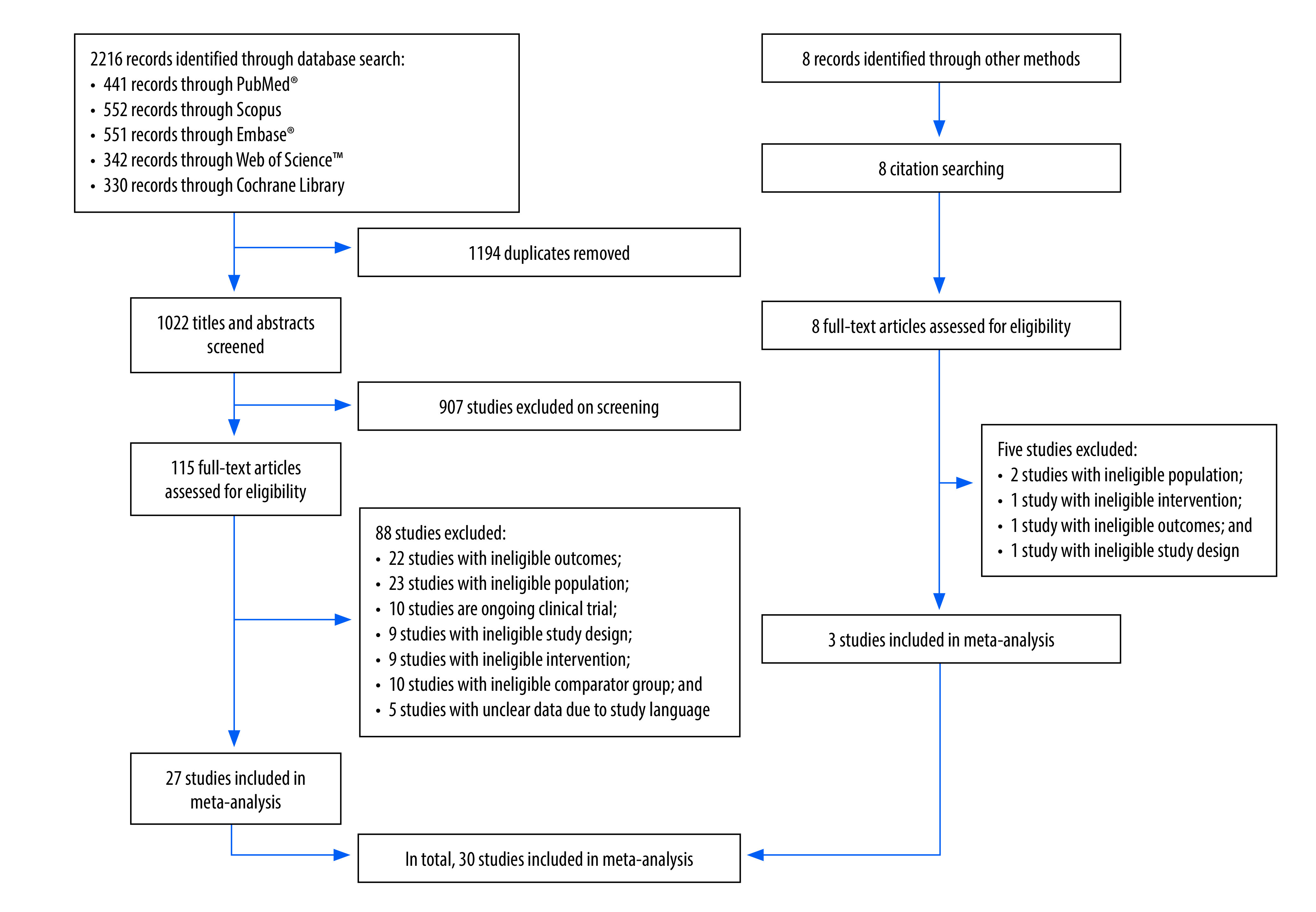
Study selection, systematic review and meta-analysis of the maternal and paternal effects of kangaroo mother care for low-birth-weight and preterm infants, 1988–2023

Our initial database search on 16 January 2023 identified 2216 records and eight additional records were identified through reference lists (Fig. 1). After removing duplicates and screening titles and abstracts, 123 articles were selected for full text review. Subsequently, 30 relevant trials from 18 countries were included in our meta-analysis (Table 1; available at https://www.who.int/publications/journals/bulletin/).^20^^–^^49^ In total, the trials reported on the effect of kangaroo mother care on the mothers and fathers of 7719 preterm or low-birth-weight infants. Eighteen trials were randomized controlled trials,^20^^–^^22^^,^^28^^–^^30^^,^^34^^–^^36^^,^^39^^,^^41^^–^^44^^,^^46^^–^^49^ whereas 12 were quasi-randomized trials (Box 2).^23^^–^^27^^,^^31^^–^^33^^,^^37^^,^^38^^,^^40^^,^^45^ There was a low risk of bias in seven trials (all randomized controlled trials),^36^^,^^41^^,^^42^^,^^44^^,^^46^^–^^48^ some concern about bias in eight,^20^^,^^29^^,^^35^^,^^39^^,^^40^^,^^43^^,^^45^^,^^49^ and a high risk of bias in 15.^21^^–^^28^^,^^30^^–^^34^^,^^37^^,^^38^ Details of our findings on bias are available from the online repository.^50^ Eight trials were conducted in low- or lower-middle-income countries,^4^^,^^28^^,^^32^^,^^42^^,^^45^^–^^47^ eight in upper-middle-income countries,^21^^,^^27^^,^^30^^,^^39^^,^^40^^,^^43^^,^^48^^,^^49^ and 14 in high-income countries.^20^^,^^22^^–^^26^^,^^29^^,^^31^^,^^33^^–^^37^^,^^41^

**Table 1:** Trials included, meta-analysis of the maternal and paternal effects of kangaroo mother care for low-birth-weight and preterm infants, 1988–2023

Author, year	Country	Trial design	Intervention group				Control group			Outcome (assessment tool)	Time of outcome assessment after childbirth	Outcome and effect size^a^
			No. preterm or low-birth-weight infants^b^	Mean gestational age (weeks)	Mean birth- weight (g)		No. preterm or low-birth-weight infants^b^	Mean gestational age (weeks)	Mean birth weight (g)			
Whitelaw et al., 1988^20^	United States	RCT	35	29.1	1152		36	29.5	1135	Mother’s confidence and positive feelings towards her baby (mother’s interview and a six-point scale)	120 days	(i) early skin-to-skin contact was no more effective than other interventions and general support in improving the mother’s confidence and positive feelings; (ii) at 6 months, infants in the intervention arm, who had skin-to-skin contact, cried significantly less than those in the control arm; and (iii) the duration of lactation was longer in the intervention arm (which is important for very-low-birth-weight infants, especially in developing countries where lactation is vital)
Tessier et al., 1998^21^	Colombia	RCT	246	33.1	1660		242	33.7	1736	(i) Mother’s attachment behaviour, including the mother’s feelings, perceptions of her premature birth experience, sense of competence, feelings of worry and stress and perception of social support (NCAST); and (ii) mother’s and child’s responsivity to each other during breastfeeding (NCAST)	9 months	(i) Skin-to-skin contact during kangaroo mother care changed mothers’ perceptions of their infants (i.e. through a subjective bonding effect); (ii) kangaroo mother care increased mothers’ feeling of competence in stressful situations when the infant had to remain in hospital longer (i.e. resilience effect); and (iii) mothers in the intervention arm felt less supported in stressful situations (i.e. isolation effect, which could be countered by social support)
Roberts et al., 2000^22^	Australia	RCT	16	31.7	1562		14	31.2	1481	Parental stress (revised PSS-NICU)	The later of 6 weeks after discharge or 3 months after birth	(i) No significant difference in mean PSS-NICU score between kangaroo mother care and control groups; and (ii) mothers exhibited moderate or high stress on all four subscales (i.e. nursery environment, infant appearance, relationship with infant, and staff behaviour and communication)
Feldman et al., 2002^23^	Israel	Quasi-randomized trial	73	30.4	1245.9		73	30.8	1289.9	(i) Depression (BDI); (ii) mother–infant interactions (Mother–Newborn Coding System); and (iii) home environment (HOME tool)	1 month	Depression score: intervention arm mean = 6.14 (SD: 4.44) and control arm mean = 7.06 (SD: 4.48); Mother–infant attachment score: intervention arm mean = 98.64 (SD: 2.72) and control arm mean = 97.00 (SD: 3.11); (i) After kangaroo mother care, interactions were more positive at 37 weeks’ gestational age, with mothers showing more positive affect, more touching and adaptation to infant cues and infants showing more alertness and less gaze aversion; (ii) mothers reported less depression and perceived infants as less abnormal; (iii) at 3 months, mothers and fathers in the intervention arm were more sensitive and provided a better home environment; and (iv) at 6 months, mothers in the intervention arm were more sensitive and infants scored higher on the Bayley Mental Developmental Index
Feldman et al., 2003^24^	Israel	Quasi-randomized trial	73	30.3	1245.9		73	30.8	1289.9	(i) Parenting stress (PSI-SF); (ii) parental competence and satisfaction (PCSS); (iii) home environment (HOME tool); (iv) mother–infant interaction (videotape assessment); and (v) father–infant interaction (videotape assessment)	1 month	In the intervention arm compared with the control arm: (i) maternal and paternal affectionate touching of infants and spouses was more frequent; and (ii) spouses remained in closer proximity to the infant, which was conducive to mutual gaze and touching during triadic play (touching is a constituent part of coregulatory parent–infant and triadic systems and maternal contact affects mothering, co-parenting and family processes)
Miles et al., 2006^25^	United Kingdom	Quasi-randomized trial	37	28	1086		24	28	1133	(i) Maternal depression (EPDS); (ii) maternal distress (GHQ); (iii) maternal stress (STAI and GHQ); and (iv) maternal confidence (MABS)	(i) Maternal depression at 1, 4 and 12 months; (ii) maternal distress at 12 months; (iii) maternal stress at 1, 4 and 12 months; and (iv) maternal confidence at 4 months	Maternal depression score at 4 months: intervention arm mean = 6.57 (SD: 4.71) and control arm mean = 6.00 (SD: 5.09); Maternal distress score at 12 months: intervention arm mean = 4.63 (SD: 3.26) and control arm mean = 6.65 (SD: 4.37); Maternal stress score at 4 months: intervention arm mean = 31.49 (SD: 10.52) and control arm mean = 30.79 (SD: 11.94); Maternal confidence score at 4 months: intervention arm mean = 14.29 (SD: 2.82) and control arm mean = 15.00 (SD: 2.14)
Tallandini & Scalembra, 2006^26^	Italy	Quasi-randomized trial	19	30.7	1179.7		21	31.6	1459.7	(i) Maternal stress (PSI-SF); and (ii) mother–infant attachment and bonding (NCAST)	52 days	Maternal stress score: intervention arm mean = 127.80 (SD: 3.05) and control arm mean = 128.23 (SD: 2.88); Mother–infant attachment score: intervention arm mean = 51.14 (SD: 1.57) and control arm mean = 45.31 (SD: 1.49)
de Macedo et al., 2007^27^	Brazil	Quasi-randomized trial	30	31.7	1387		60	33.6	1934	Mood variation (VAMS)	2 weeks	(i) Mothers in the intervention arm reported fewer occurrences of depressive states than control group mothers; (ii) mothers in the intervention arm reported feeling calmer, stronger, better coordinated and more energetic, contented, tranquil, quick-witted, relaxed, proficient, happy, friendly and clearheaded; and (iii) kangaroo mother care had a positive effect on mood variation among preterm mothers
Gathwala et al., 2008^28^	India	RCT	55	35.5	1690		55	35.1	1690	Maternal and child attachment and bonding (SMI)	3 months	Mother–infant attachment score: intervention arm mean = 24.46 (SD: 1.64) and control arm mean = 18.22 (SD: 1.79)
Chiu & Anderson, 2009^29^	United States	RCT	40	34.4	2257		29	34.6	2211	Mother–infant attachment and bonding (NCAST Nursing Child Assessment of Feeding Scale)	6 months	Mother–infant attachment score: intervention arm mean = 89.9 (SD: 2.68) and control arm mean = 78.2 (SD: 8.4)
Tessier et al., 2009^30^	Colombia	RCT	194	33.1	1660		144	33.7	1736	(i) Father’s involvement (Perception of Premature Birth Questionnaire – mother subscale); and (ii) home environment and quality (HOME tool)	12 months	(i) Mothers in the intervention arm created a more stimulating and better caregiving environment for their child than mothers in the control arm; (ii) there was a positive correlation between the quality of the environment and the father’s involvement; and (iii) the family environment of male infants was improved most by kangaroo mother care.
Ahn et al., 2010^31^	Republic of Korea	Quasi-randomized trial	10	32.1	1486		10	31.8	1572	(i) Maternal attachment (MAI); and (ii) postpartum depression (EPDS)	4 months	Mother–infant attachment score: intervention arm mean = 89.91 (SD: 2.68) and control arm mean = 78.20 (SD: 8.40); Postpartum depression score: intervention arm mean = 7.22 (SD: 4.04) and control arm mean = 6.31 (SD: 4.13)
Badiee et al., 2014^32^	Islamic Republic of Iran	Quasi-randomized trial	25	36	2100		25	35.9	2013	(i) Physical well-being; (ii) anxiety; (iii) social well-being; and (iv) depression (GHQ)	1 month	Depression score: intervention arm mean = 4.96 (SD: 4.24) and control arm mean = 4.96 (SD: 4.96); Anxiety score: intervention arm mean = 5.96 (SD: 3.10) and control arm mean = 9.96 (SD: 4.06); Maternal distress score: intervention arm mean = 25.63 (SD: 8.74) and control arm mean = 31.21 (SD: 12.31); Maternal physical well-being score: intervention arm mean = 8.56 (SD: 3.24) and control arm mean = 8.73 (SD: 3.72)
Feldman et al., 2014^33^	Israel	Quasi-randomized trial	73	30.4	1245.9		73	30.8	1289.9	(i) Parental depression (BDI); (ii) parental anxiety symptoms (STAI); and (iii) mother–infant attachment and bonding (videotape assessment)	6 months	Maternal depression score: intervention arm mean = 6.14 (SD: 4.44) and control arm mean = 7.06 (SD: 4.48); Maternal anxiety score: intervention arm mean = 31.47 (SD: 6.22) and control arm mean = 34.95 (SD: 8.64); Mother–infant attachment: intervention arm mean = 0.53 (SD: 0.23) and control arm mean = 0.44 (SD: 0.21)
Holditch-Davis et al., 2014^34^	United States	RCT	78	27.2	1021.7		81	27.4	1023.0	(i) Maternal depression (CES-D); (ii) maternal anxiety (STAI); (iii) post-traumatic stress (PPQ); (iv) parenting stress (PSS); and (v) worry about child’s health (Worry Index)	60 days	Anxiety: B-coefficient = −0.94 (SE: 1.4); Parenting stress: B-coefficient = −0.75 (SE: 2.35); Post-traumatic stress: B-coefficient = 0.05 (SE: 0.45); Worry about child’s health: B-coefficient = −0.6 (SE: 3.95)
Mörelius et al., 2015^35^	Sweden	RCT	23	34	2468		19	34	2512	(i) Parental stress (SPSQ); (ii) parental depression (EPDS); (iii) maternal sensitivity (ASS); and (iv) relationship between spouses (SPSQ spouse relationship problems subscale)	(i) Paternal stress at 1 and 4 months; (ii) paternal depression at 4 months; (iii) maternal sensitivity at 1 month; and (iv) relationship between spouses at 1 and 4 months	Maternal stress score at 1 month: intervention arm mean = 2.30 (SD: 0.46) and control arm mean = 2.30 (SD: 0.64); Maternal depression score at 4 months: intervention arm mean = 3.62 (SD: 4.41) and control arm mean = 3.97 (SD: 4.93); Paternal depression score: intervention arm mean = 3.80 (95% CI: 2.66 to 5.91) and control arm mean = 4.54 (95% CI: 4.22 to 10.11); Paternal stress at 1 month: intervention arm median = 2.3 (IQR: 2.10 to 2.51) and control arm median = 2.2 (IQR: 1.9 to 2.5); Maternal sensitivity score: intervention arm median = 2.00 (IQR: 1.00 to 4.25) and control arm median = 2.50 (IQR: 1.51 to 4.00); Relationship with spouse score at 1 month: intervention arm median = 2.10 (IQR: 1.51 to 2.52) and control arm median = 1.81 (IQR: 1.41 to 2.50); Relationship with spouse score at 4 months: intervention arm median = 4.51 (IQR: 2.01 to 5.25) and control arm median = 4.50 (IQR: 2.00 to 6.00)
Samra et al., 2015^36^	United States	RCT	20	35	2493		20	35.5	2693	Maternal stress (PSS-NICU)	14 days	Maternal stress score: intervention arm mean = 2.55 (SD: 0.95) and control arm mean = 2.70 (SD: 0.90)
Cho et al., 2016^37^	Republic of Korea	Quasi-randomized trial	20	33.7	1600		20	33	1442	(i) Mother–infant attachment (MAI); and (ii) maternal stress (PSS-NICU)	21 days	Mother–infant attachment score: intervention arm mean = 4.74 (SD: 0.28) and control arm mean = 4.48 (SD: 0.39); Maternal stress score: intervention arm mean = 3.76 (SD: 0.23) and control arm mean = 4.40 (SD: 0.33)
Zahedpasha et al., 2018^38^	Islamic Republic of Iran	Quasi-randomized trial	25	36	2400		25	36	2400	(i) Maternal distress (GHQ); (ii) maternal anxiety (GHQ); and (iii) maternal depression (GHQ)	7 days	Maternal distress score: intervention arm mean = 6.51 (SD: 13.56) and control arm mean = 8.89 (SD: 20.91); Maternal anxiety score: intervention arm mean = 1.50 (SD: 3.70) and control arm mean = 3.39 (SD: 5.67); Maternal depression score: intervention arm mean = 1.32 (SD: 1.03) and control arm mean = 2.31 (SD: 2.72)
Coşkun & Günay, 2020^39^	Türkiye	RCT	42	33	1500		42	33	1500	Stress (PSS-NICU)	3 weeks	Stress score: intervention arm mean = 41.22 (SD: 3.90) and control arm mean = 40.41 (SD: 4.91)
Kurt et al., 2020^40^	Türkiye	Quasi-randomized trial	30	33	1985.8		30	32.8	2028.8	Maternal–child attachment and bonding (MAI)	5 days	Mother–infant attachment score: intervention arm mean = 35.03 (SD: 5.54) and control arm mean = 29.87 (SD: 4.66)
Mehler et al., 2020^41^	Germany	RCT	44	29	1250		43	29	1170	(i) Maternal depression (CES‐D); (ii) maternal stress (PSI); and (iii) parental bonding (PBQ)	6 months	Maternal depression at discharge (RR: 1.0; 95% CI: 0.4 to 2.5); Maternal depression at 6 months (RR: 0.5; 95% CI: 0.1 to 2.8); Maternal stress (OR: 1.00; 95% CI: 1.0 to 1.11); Mother–infant attachment and bonding (OR: 1.1; 95% CI: 1.0 to 1.3)
Taneja et al., 2020^42^	India	RCT	273	35.6	2051		271	35.7	2066	(i) Maternal depression (PHQ-9); (ii) maternal self-efficacy score; and (iii) home environment (PROCESS questionnaire)	6 weeks and 6 months	Maternal depression score at 6 weeks: intervention arm mean = 2.02 (SD: 3.04) and control arm mean = 2.11 (SD: 2.6); Maternal depression score at 6 months: intervention arm mean = 0.54 (SD: 1.4) and control arm mean = 0.55 (SD: 1.3); Maternal self-efficacy score: intervention arm mean = 37.13 (SD: 2.7) and control arm mean = 37.25 (SD: 2.7); Maternal self-efficacy score (MD: 0.14; 95% CI: −0.34 to 0.62); PROCESS score: intervention arm mean = 123.01 (SD: 16.6) and control arm mean = 125.02 (SD: 16.5); Multivariable analysis showed that the intervention had no significant effect on PROCESS scores
Wang et al., 2020^43^	China	RCT	114	36	2400		116	36	2400	(i) Maternal stress (PSI-SF); and (ii) maternal anxiety (PSS-NICU)	14 days	Maternal stress score: intervention arm mean = 79.09 (SD: 8.02) and control arm mean = 89.46 (SD: 8.74); Maternal anxiety score: intervention arm mean = 2.57 (SD: 0.81) and control arm mean = 3.47 (SD: 0.93)
Arya et al., 2021^44^^c^	Ghana, India, Malawi, Nigeria and the United Republic of Tanzania	Multi-site RCT	1276	32.6	1500		1231	32.6	1500	(i) Moderate-to-severe postpartum depression (CES-D); and (ii) maternal satisfaction (interview)	28 days	Moderate-to-severe postpartum depression (RR: 0.23; 95% CI: 0.05 to 1.14); Maternal satisfaction: intervention arm mean = 9.21 (SD: 1.00) and control arm mean = 9.11 (SD: 1.20)
Gholami et al., 2021^45^	Islamic Republic of Iran	Quasi-randomized trial	30	32.7	1456.3		60	26.1	1388.0	Overt and covert anxiety (SSOVAQ)	1 month	Overt anxiety score: intervention arm mean = 5.96 (SD: 3.10) and control arm mean = 9.96 (SD: 4.06); Covert anxiety score: intervention arm mean = 7.65 (SD: 5.11) and control arm mean = 8.02 (SD: 2.32)
Karimi et al., 2021^46^	Islamic Republic of Iran	RCT	30	31.7	1456.3		30	31.5	1396.3	(i) Depression, anxiety and stress (DASS-21); and (ii) emotion-focused coping and problem-solving-based coping (CRI-A)	1 month	Depression score: intervention arm mean = 10.47 (SD: 4.16) and control arm mean = 17.83 (SD: 5.57); Anxiety score: intervention arm mean = 9.57 (SD: 3.64) and control arm mean = 15 (SD: 4.34); Stress score: intervention arm mean = 11.40 (SD: 3.87) and control arm mean = 19.67 (SD: 6.23); Emotion-focused coping score: intervention arm mean = 56.43 (SD: 6.97) and control arm mean = 47.37 (SD: 8.91); Problem-solving-based coping score: intervention arm mean = 8.87 (SD: 11.81) and control arm mean = 16.87 (SD: 2.93)
Sinha et al., 2021^47^	India	RCT	974	35.8	2100		852	35.8	2100	Maternal depression (PHQ-9)	28 days	Maternal depression score: intervention arm mean = 3.62 (SD: 4.4) and control arm mean = 3.97 (SD: 4.92); Maternal depression (RR: 0.79; 95% CI: 0.62 to 1.01)
Chen et al., 2022^48^	China	RCT	63	32	1987		63	32	1986	(i) Psychological problems (SCL-90); and (ii) insomnia (AIS)	4 weeks	Stress score: intervention arm mean = 127.85 (SD: 19.99) and control arm mean = 147.31 (SD: 18.76); Sleep quality score: intervention arm mean = 6.41 (SD: 2.69) and control arm mean = 8.98 (SD: 2.34)
Erduran & Yaman Sözbir (2022)^49^	Türkiye	RCT	30	36	2300		30	36	2400	(i) Depression (EPDS); and (ii) mother–infant attachment and bonding (MAI)	1 week	Depression score: intervention arm mean = 8.43 (SD: 6.62) and control arm mean = 8.29 (SD: 4.67); Mother–infant attachment score: intervention arm mean = 98.64 (SD: 2.72) and control arm mean = 97 (SD: 3.11)

AIS: Athens Insomnia Scale; ASS: Ainsworth’s Sensitivity Scale; BDI: Beck Depression Inventory; CES-D: Centre for Epidemiologic Studies Depression Scale; CI: confidence interval; CRI-A: Coping Responses Inventory–Adult; DASS-21: Depression, Anxiety and Stress Scale–21 items; EPDS: Edinburgh Postnatal Depression Scale; GHQ: General Health Questionnaire; HOME: Home Observation for Measurement of the Environment; IQR: interquartile range; MABS: Mother and Baby Scale; MAI: Maternal Attachment Inventory; MD: mean difference; NCAST: Nursing Child Assessment Satellite Training; OR: odds ratio; PBQ: Parental Bonding Questionnaire; PCSS: Parental Competence and Satisfaction Scale; PHQ-9: Patient Health Questionnaire; PPQ: Perinatal Post Traumatic Stress Disorder Questionnaire; PROCESS: Pediatric Review of Children’s Environmental Support and Stimulation; PSI-SF: Parenting Stress Index-Short Form; PSS: Parental Stress Scale; PSS-NICU: Parental Stressor Scale–Neonatal Intensive Care Unit; RCT: randomized controlled trial; RR: relative risk; SCL-90: Symptom Checklist-90; SD: standard deviation; SE: standard error; SMI: Structured Maternal Interview; SPSQ: Swedish Parenthood Stress Questionnaire; SSOVAQ: Spielberger Standard Overt and Covert Anxiety Questionnaire; STAI: State–Trait Anxiety Inventory; VAMS: Visual Analogue Mood Scale.

^a^ The RR of, or the OR for, the outcome in the kangaroo mother care arm versus the no- kangaroo mother care arm is reported.

^b^ Preterm was defined as under 37 weeks’ gestation and a low birth weight was defined as under 2500 g.

^c^ This study compared immediate kangaroo mother care with delayed kangaroo mother care until the infant was stable on incubator care.

Box 2 **Trial characteristics, meta-analysis of the maternal and paternal effects of kangaroo mother care for low-birth-weight and preterm infants, 1988–2023**

**General trial characteristics**

- No. of trials in meta-analysis: 30
- No. of infants in meta-analysis: 7719
- Countries covered by trials: Australia, Brazil, China, Colombia, Germany, Ghana, India, Islamic Republic of Iran, Israel, Italy, Malawi, Nigeria, Republic of Korea, Sweden, Türkiye, United Kingdom, United Republic of Tanzania, United States

**Publication year range: 1988–2023**

**Trial design**

- No. of randomized controlled trials: 18
- No. of quasi-randomized trials: 12

**Infant characteristics**

- No. of pretermor low-birth-weight infants:^a^ 7719
- Mean gestational age, weeks: kangaroo mother care group: 32.6; control group: 32.6
- Mean birth weight, g: kangaroo mother care group: 1703; control group: 1733

**Country income group**

- Low income: one country
- Lower-middle income: five countries
- Upper-middle income: four countries
- High income: eight countries

**Trial intervention and comparator care**

- Kangaroo mother care versus standard or routine care: 20 trials
- Kangaroo mother care versus incubator care: 10 trials

**Kangaroo mother care characteristics**

- Total duration of kangaroo mother care in days, mean (SD): 23.7 (17.3)
- Total duration of kangaroo mother care in days, median (IQR): 23.0 (13.3 to 28.0)
- Kangaroo mother care practiced daily: 27 studies
- Kangaroo mother care practiced 3 to 5 days a week: three studies
- Duration of skin-to-skin contact per day in hours, mean (SD): 5.22 (8.23)
- Duration of skin-to-skin contact per day in hours, median (IQR): 1.0 (0.9 to 7.5)

**Place kangaroo mother care initiated**

- Hospital: 28 studies
- Community: two studies

**Kangaroo mother care provider**

- Mother alone: 24 studies
- Mother with father or other caregiver: six studies

IQR: interquartile range; SD: standard deviation.^a^ Preterm was defined as under 37 weeks’ gestation and a low birth weight was defined as under 2500 g.

In 20 trials, the control group of preterm and low-birth-weight infants received standard or routine care through a government health system,^22^^,^^25^^,^^26^^,^^28^^,^^31^^,^^34^^–^^43^^,^^45^^–^^49^ whereas, in 10, they underwent incubator care (Box 2).^20^^,^^21^^,^^23^^,^^24^^,^^27^^,^^29^^,^^30^^,^^32^^,^^33^^,^^44^ Across all studies, the mean duration of kangaroo mother care was 23.7 days (standard deviation, SD: 17.3) and the median duration was 23.0 days (interquartile range, IQR: 13.3–28.0). The mean daily duration of skin-to-skin contact was 5.22 hours (SD: 8.23) and the median was 1.0 hours (IQR: 0.9 to 7.5). In 80% (24/30) of trials, the mother was the primary giver of kangaroo mother care.^4^^,^^20^^,^^22^^,^^25^^–^^29^^,^^31^^,^^32^^,^^34^^,^^36^^,^^37^^,^^39^^–^^43^^,^^45^^–^^49^ Kangaroo mother care was initiated in the community in only 6.7% (2/30) of trials.^42^^,^^47^ The mean birth weight in the intervention and control groups was 1703 g and 1733 g, respectively, and the mean gestational age was 32.6 weeks in both groups.

### Primary outcomes

**Fig. 2:**
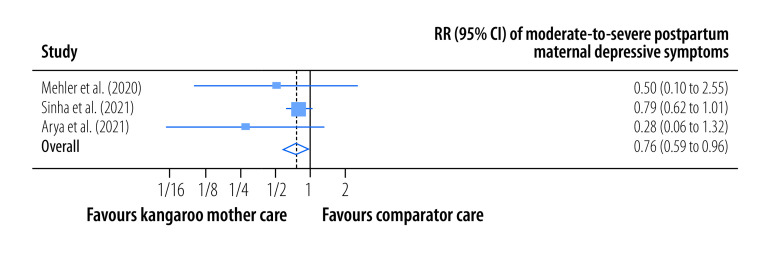
Risk of moderate-to-severe postpartum maternal depressive symptoms, meta-analysis of the maternal and paternal effects of kangaroo mother care for low-birth-weight and preterm infants, 1988–2023

CI: confidence interval; RR: relative risk.

Note: The Arya et al. (2021) trial compared immediate kangaroo mother care with delayed kangaroo mother care until the infant was stable on incubator care. Postpartum depressive symptoms were assessed a median of 44 (interquartile range: 28 to 59) days after childbirth.

**Fig. 3:**
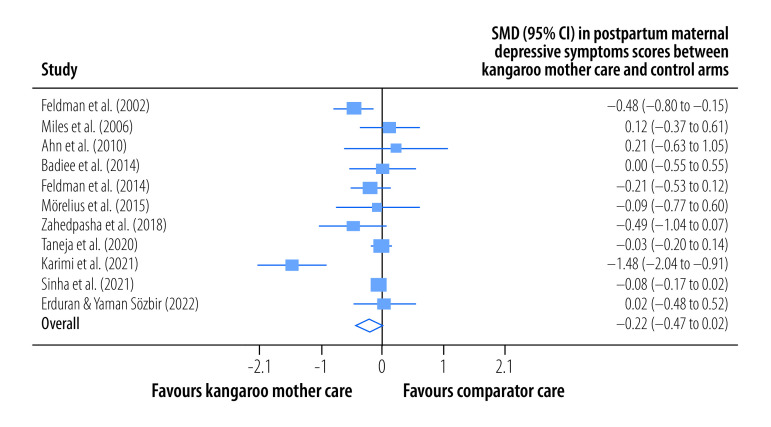
Effect of kangaroo mother care on postpartum maternal depressive symptoms, meta-analysis of the maternal and paternal effects of kangaroo mother care for low-birth-weight and preterm infants, 1988–2023

CI: confidence interval; SMD: standardized mean difference.

Note: The SMD was calculated as the mean difference between the mean outcome scores in the kangaroo mother care and control groups in each trial divided by their respective standard deviations. Postpartum depressive symptom scores were assessed a median of 30 (interquartile range: 7 to 180) days after childbirth.

Summary statistics for the effect of kangaroo mother care on primary study outcomes are shown in Table 2. The pooled RR for moderate-to-severe postpartum depressive symptoms in mothers whose infants received kangaroo mother care compared with mothers whose infants did not was 0.76 (95% CI: 0.59 to 0.96; *I^2^* = 0%; three trials; 4399 participants; high certainty) at the latest follow-up a median of 44 days (IQR: 28–59) after childbirth (Fig. 2). Moreover, the overall, pooled, mean postpartum maternal depressive symptoms score was lower among mothers in the kangaroo mother care group versus the control group (SMD: −0.22; 95% CI: −0.47 to 0.02; *I^2^* = 83.41%; 11 trials; 3000 participants; low certainty) at the latest follow-up a median of 30 days (IQR:7–180) after childbirth (Fig. 3). 

**Table 2:** Key maternal health outcomes, meta-analysis of the maternal and paternal effects of kangaroo mother care for low-birth-weight and preterm infants, 1988–2023

Outcome	No. trials	No. infant participants	Mean (SD) duration of follow-up, days after birth	Median (IQR) duration of follow-up, days after birth	Certainty of the evidence^a^	Effect size^b,c^ (95% CI)
Moderate-to-severe postpartum maternal depressive symptoms	3 RCTs	4399	43.5 (15.5)	44 (28 to 59)	High	RR: 0.76 (0.59 to 0.96)^d^
Postpartum maternal depressive symptoms score	5 RCTs and 6 QRTs	3000	82.4 (104.1)	30 (7 to 180)	Low^e^	SMD: −0.22 (−0.47 to 0.02)
Maternal stress score	7 RCTs and 3 QRTs	794	78.9 (126.8)	30 (19 to 74)	Low^f^	SMD: −0.82 (−1.32 to −0.32)
Maternal anxiety score	1 RCT and 5 QRTs	463	62.0 (71.4)	29 (7 to 120)	Low^g^	SMD: −0.62 (−1.01 to −0.23)
Maternal distress score	2 QRTs	100	7 (ND)	7 (ND)	Very low^h^	MD: −4.71 (−9.77 to 0.35)
Mother–infant attachment and bonding score	3 RCTs and 6 QRTs	450	110.3 (137.2)	52 (14 to 180)	Low^i^	SMD: 1.19 (0.27 to 2.10)

CI: confidence interval; IQR: interquartile range; MD: mean difference; ND: not determined; QRT: quasi-randomized trial; RCT: randomized controlled trial; RR: relative risk; SD: standard deviation; SMD: standardized mean difference.

^a^ The certainty of the evidence for the pooled estimates was assessed using the GRADE approach.^14^

^b^ The relative risk (RR) is the risk of the outcome in the kangaroo mother care arm versus the control arm with no kangaroo mother care.

^c^ The standardized mean difference (SMD) was calculated as the mean difference between the mean outcome scores in the kangaroo mother care and control groups in each trial divided by their respective standard deviations.

^d^ This figure corresponds to 14 (95% confidence interval: 2 to 25) fewer cases of moderate-to-severe postpartum maternal depressive symptoms per 1000 mothers.

^e^ The certainty was downgraded two levels because there was a serious risk of bias and serious inconsistency (i.e. *I^2^* = 83.41%; Egger’s *P*-value: 0.0196).

^f^ The certainty was downgraded two levels because there was a serious risk of bias and serious inconsistency (*I^2^* = 90.49%; Egger’s *P*-value: 0.0145).

^g^ The certainty was downgraded two levels because there was a serious risk of bias and serious inconsistency (*I^2^* = 74.83%; Egger’s *P*-value: 0.1074).

^h^ The certainty was downgraded three levels because there was a serious risk of bias, serious indirectness (i.e. small sample size) and serious imprecision (i.e. wide confidence intervals).

^i^ The certainty was downgraded two levels because there was a serious risk of bias and serious inconsistency (*I^2^* = 96.06%; Egger’s *P*-value: 0.012).

**Fig. 4:**
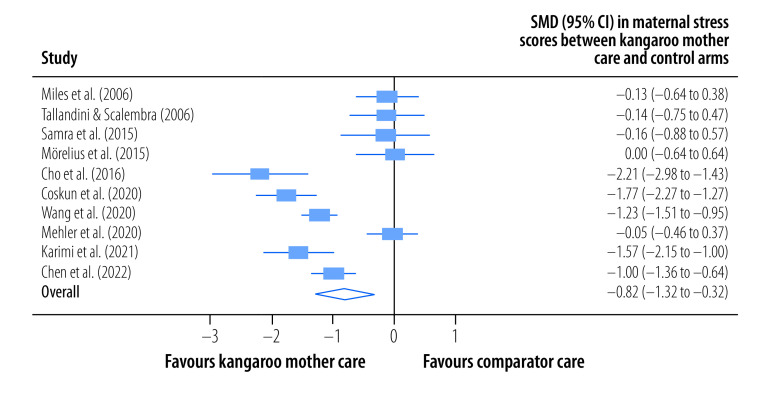
Effect of kangaroo mother care on maternal stress, meta-analysis of the maternal and paternal effects of kangaroo mother care for low-birth-weight and preterm infants, 1988–2023

CI: confidence interval; SMD: standardized mean difference.

Note: The SMD was calculated as the mean difference between the mean outcome scores in the kangaroo mother care and control groups in each trial divided by their respective standard deviations. Maternal stress scores were assessed a median of 30 (interquartile range: 19 to 74) days after childbirth.

**Fig. 5:**
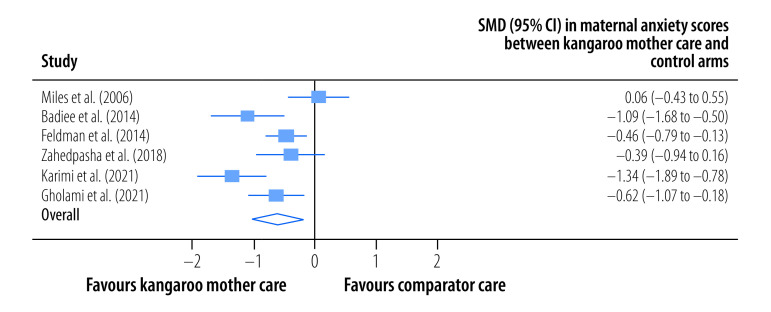
Effect of kangaroo mother care on maternal anxiety, meta-analysis of the maternal and paternal effects of kangaroo mother care for low-birth-weight and preterm infants, 1988–2023

CI: confidence interval; SMD: standardized mean difference.

Note: The SMD was calculated as the mean difference between the mean outcome scores in the kangaroo mother care and control groups in each trial divided by their respective standard deviations. Maternal anxiety scores were assessed a median of 29 (interquartile range: 7 to 120) days after childbirth.

**Fig. 6:**
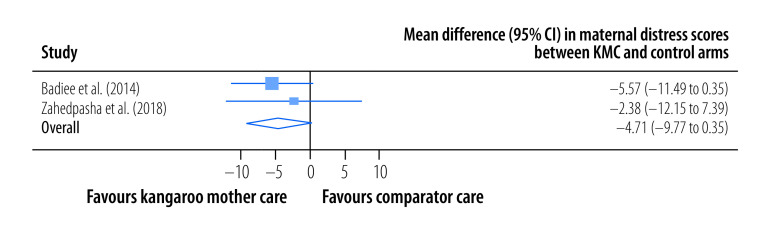
Effect of kangaroo mother care on maternal distress, meta-analysis of the maternal and paternal effects of kangaroo mother care for low-birth-weight and preterm infants, 1988–2023

The pooled, mean maternal stress score was significantly lower among mothers in the kangaroo mother care group than among those in the control group (SMD: −0.82; 95% CI: −1.32 to −0.32; *I^2^* = 90.49%; 10 trials; 794participants; low certainty) at the latest follow-up a median of 30 days (IQR: 19–74) after childbirth (Fig. 4). In addition, the pooled, mean maternal anxiety score was significantly lower among mothers in the kangaroo mother care group (SMD: −0.62; 95% CI: −1.01 to −0.23; *I^2^* = 74.83%; six trials; 463 participants; low certainty) at the latest follow-up a median of 29 days (IQR: 7–120) after childbirth (Fig. 5). The pooled mean difference in maternal distress score, as assessed using the general health questionnaire,^51^ between mothers in the kangaroo mother care arm and those in the control arm was −4.71 (95% CI: −9.77 to 0.35; *I^2^* = 0%; two trials; 100 participants; very low certainty) at the latest follow-up a median of 7 days after childbirth (Fig. 6).

CI: confidence interval.

Note: Maternal distress was assessed using the General Health Questionnaire a median of 7 days after childbirth.

### Secondary outcomes

**Fig. 7:**
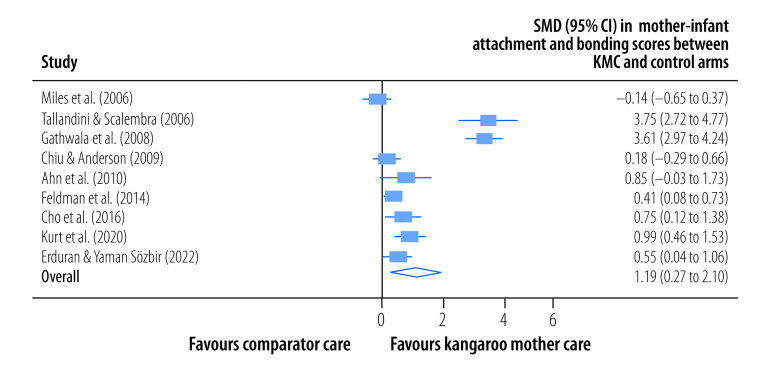
Effect of kangaroo mother care on mother–infant attachment and bonding, meta-analysis of the maternal and paternal effects of kangaroo mother care for low-birth-weight and preterm infants, 1988–2023

CI: confidence interval; SMD: standardized mean difference.

Note: The SMD was calculated as the mean difference between the mean outcome scores in the kangaroo mother care and control groups in each trial divided by their respective standard deviations. Mother–infant attachment and bonding scores were assessed a median of 52 (interquartile range: 14 to 180) days after childbirth.

The pooled, mean, mother–infant attachment and bonding score was significantly higher for mothers in the kangaroo mother care arm than for those in the control arm (SMD: 1.19; 95% CI: 0.27 to 2.10; *I^2^* = 96.06%; nine trials; 450 participants; low certainty) at the latest follow-up a median of 52 days (IQR: 14–180) after childbirth (Fig. 7). Three trials reported the effect of kangaroo mother care on paternal health outcomes (Table 3). First, a randomized controlled trial in Sweden with 37 participants reported that 7.1% of fathers in the kangaroo mother care arm had depressive symptoms compared to 8.3% in the control arm but the difference was not significant.^35^ That trial also reported a decrease in relationship problems with spouses. However, no difference in paternal stress scores was observed between the groups. Second, a quasi-randomized study in Israel with 146 participants reported that fathers in the kangaroo mother care group were more sensitive, less intrusive and showed higher reciprocity than those in the control group.^24^ Third, a randomized controlled trial in Canada with 338 participants found that kangaroo mother care had a positive impact on the home environment and was positively correlated with the father's involvement in child care.^30^ Some other trials reported on additional maternal health outcomes, such as sensitivity, mood variance, confidence, satisfaction, duration of lactation, coping skills and sleep quality. Details are available from the online repository.^50^ We did not find any reports on maternal or paternal physical health outcomes.

**Table 3:** Studies on paternal health outcomes, meta-analysis of the maternal and paternal effects of kangaroo mother care for low-birth-weight and preterm infants, 1988–2023

Author, year	Country	Trial design	Trial participants	Intervention	Outcome (assessment tool)	Findings	Interpretation
Mörelius et al., 2015^35^	Sweden	RCT	37 infants (18 intervention arm and 19 control arm)	Kangaroo mother care was initiated in hospital and provided daily for 12 days by the mother and father	(i) Paternal stress 4 months after birth (SPSQ); (ii) paternal depression 4 months after birth (EPDS); and (iii) relationship with spouse 4 months after birth (SPSQ spouse relationship subscale)	Paternal stress score: intervention arm median = 2.3 (IQR: 2.1 to 2.5) and control arm median = 2.2 (IQR 1.9 to 2.5); Paternal depression score: intervention arm mean = 3.80 (95% CI: 2.66 to 5.91) and control arm mean = 4.54 (95% CI: 4.22 to 10.1); 7.1% of fathers in the intervention arm had depressive symptoms (i.e. an EPDS score > 9) versus 8.3% in the control arm; Spouse relationship subscale score: intervention arm median = 2.0 (IQR: 1.4 to 2.0) and control arm median = 2.6 (IQR 2.0 to 3.0)	(i) Paternal stress scores were similar in the kangaroo mother care and control arms; (ii) the proportion of fathers with depressive symptoms was lower in the intervention arm but the difference was not significant; and (iii) kangaroo mother care appeared to decrease fathers’ experience of spouse relationship problems
Feldman et al., 2003^24^	Israel	Quasi-randomized trial	146 infants (73 intervention arm and 73 control arm)	Kangaroo mother care was initiated in hospital and provided daily for 14 days by the mother and father	Father–infant interaction 6 months after birth (videotape assessment): parameters included paternal sensitivity, paternal intrusiveness, infant positive affect, infant negative emotionality, infant initiation of interaction and involvement, and dyadic reciprocity	Paternal sensitivity score: intervention arm mean = 4.19 (SD: 0.58) and control arm mean = 3.76 (SD: 0.78; *P* < 0.05); Paternal intrusiveness score: intervention arm mean = 2.02 (SD: 0.98) and control arm mean = 2.74 (SD: 0.89; *P* < 0.05); Infant positive affect score: intervention arm mean = 3.74 (SD: 0.78) and control arm mean = 3.65 (SD: 0.79; *P* > 0.05); Infant negative emotionality score: intervention arm mean = 1.27 (SD: 0.81) and control arm mean = 1.56 (SD: 0.86; *P* < 0.05); Infant initiation of interaction and involvement score: intervention arm mean = 1.97 (SD: 0.80) and control arm mean = 2.11 (SD: 0.57; *P* > 0.05); Dyadic reciprocity score: intervention arm mean = 3.56 (SD: 1.02) and control arm mean = 3.06 (SD: 1.17; *P* < 0.05)	(i) Fathers in the intervention arm were more sensitive and less intrusive than those in the control arm; (ii) infants in the intervention arm showed less negative emotionality; and (iii) dyadic reciprocity was higher in the intervention arm
Tessier et al., 2009^30^	Colombia	RCT	338 infants (194 intervention arm and 144 control arm)	Kangaroo mother care was initiated in hospital and provided daily for 14 days by the mother and father	(i) Father’s contribution to the home environment (HOME tool); and (ii) mother’s perception of father’s involvement in the home environment (classified as low or high)	Mothers perceived fathers in the intervention arm to be more involved in the home environment, which positively affected the families of both boys and girls (*P* < 0.01)	The home environment was more stimulating and involved greater caregiving in the intervention arm than in the traditional care arm, which was positively correlated with the father’s involvement

CI: confidence interval; EPDS: Edinburgh Postnatal Depression Scale; HOME: Home Observation for Measurement of the Environment; IQR: interquartile range; ND: not determined; RCT: randomized controlled trial; SD: standard deviation; SPSQ: Swedish Parenthood Stress Questionnaire.

The subgroup analysis indicated that kangaroo mother care, whether given by the mother alone or by the mother and father together, can reduce maternal depressive symptoms and anxiety. Details are available from the online repository.^50^ The beneficial effects of kangaroo mother care on maternal mental health outcomes seemed to be most prominent in the first 6 months after birth, and the effect was greater in lower-middle-income countries than high-income countries.

## Discussion

Our meta-analysis included 30 trials from 18 countries that evaluated the effect of kangaroo mother care on the health of the mothers and fathers of 7719 preterm or low-birth-weight infants. We found high-certainty evidence that kangaroo mother care can substantially reduce the risk of moderate-to-severe, postpartum, maternal depressive symptoms. In addition, there was low-certainty evidence for a small or moderate decrease in postpartum, maternal depressive symptoms of any severity and in stress and anxiety, and for a small or moderate increase in mother–infant attachment and bonding. Very-low-certainty evidence from two trials indicated that kangaroo mother care reduced maternal distress, and evidence from three trials suggested it improved father–infant interactions. No substantial effect was observed on paternal depression or stress, or on maternal or paternal physical health.

Our findings substantiate evidence from previous systematic reviews of the effect of kangaroo mother care on maternal health. A systematic review published in 2014 reported inconclusive findings on whether kangaroo mother care for preterm or low-birth-weight infants ameliorated negative maternal mood or promoted positive maternal and paternal interactions with the infant.^11^ However, that review did not perform a meta-analysis. In 2019, a systematic review and meta-analysis reported that kangaroo mother care for preterm or low-birth-weight infants was associated with a 1.04% reduction in the pooled standardized mean depression score (*I^2^* = 82%; four trials) in mothers relative to the control group.^52^ In addition, a 2021 meta-analysis reported that kangaroo mother care for premature infants significantly reduced the level of maternal anxiety (SMD: −0.72; 95% CI: −1.08 to −0.35; *I^2^* = 75%; six trials) and maternal stress (SMD: −0.84; 95% CI: −1.59 to −0.09; *I^2^* = 90%; four trials) compared with no kangaroo mother care.^13^ However, that meta-analysis did not include studies involving low-birth-weight infants born at full term, and did not report other maternal or paternal health outcomes. Moreover, no previous meta-analysis assessed the overall quality of the evidence.

Our meta-analysis contributes to the existing literature by providing an up-to-date synthesis of the evidence from trials that evaluated the effect of kangaroo mother care for preterm and low-birth-weight infants on maternal or paternal health outcomes. We report pooled estimates for a wide range of outcomes, including postpartum maternal depressive symptoms, stress, anxiety, distress, sensitivity, mood variance and sense of competence and mother–infant attachment and bonding. Full details of our findings on the maternal sense of competence, with an interpretation, are available from the online repository.^50^ Furthermore, the inclusion of 7719 infants means we were able to report primary outcomes with high statistical power, and coverage of a variety of low-, middle- and high-income countries means our findings may be widely generalizable. In addition, our evaluation of the certainty of the evidence in a quality assessment may be useful for framing future recommendations.

Although we did not find eligible studies on the effect of kangaroo mother care for preterm or low-birth-weight infants on maternal physical health outcomes, it is noteworthy that a meta-analysis from 2019 (six trials; 498 participants)^53^ found that mother–infant skin-to-skin contact immediately after delivery of full-term infants with a normal birth weight was associated with a shorter third stage of labour compared with no skin-to-skin contact (mean difference: −1.33 minutes; 95% CI: −2.31 to −0.36).

Biologically, the beneficial effect of kangaroo mother care on the mother’s mental health (i.e. less postpartum depression, anxiety and stress) could be explained by better mother–infant bonding and complex physiological mechanisms, potentially through increased oxytocin release.^54^ It has been observed that mothers who have a prolonged separation from their infants due to neonatal intensive care admission or another issue are more likely to develop negative emotions such as despair and feelings of reduced competence and confidence.^52^^,^^55^ Kangaroo mother care provides the mother and infant with an opportunity for close contact, which helps the mother gain self-confidence in caring for her premature infant.^40^^,^^56^^,^^57^ Hence, the mother is more responsive to her child’s needs, which improves the quality of the infant’s attachment to its mother and family.^32^ In addition, kangaroo mother care helps the baby recognize its parents. In the studies included in our meta-analysis, the duration of skin-to-skin contact varied substantially; the mean was 5.2 hours per day over 23 days in the postpartum period. Kangaroo mother care has also been associated with improved breastfeeding,^58^^,^^59^ which is another trigger for oxytocin release and could be an alternative explanation for better health outcomes in mothers practicing kangaroo mother care. It is possible that the positive effect of skin-to-skin contact on mother–infant bonding may have facilitated the initiation of breastfeeding and encouraged exclusive breastfeeding.

The main limitations of our analysis were the high between-study heterogeneity and high risk of bias in 50% (15/30) of studies included. Although a predefined subgroup analysis was unable to identify the reason for the heterogeneity, it is possible the use of different assessment tools and time-points for quantifying mental health outcomes may have contributed. Nonetheless, the findings of the subgroup analysis should be interpreted with caution because the subgroups contained relatively few studies or participants and, consequently, effect size estimates may be imprecise.

In conclusion, kangaroo mother care is known to benefit preterm and low-birth-weight infants. Our study provides comprehensive, up-to-date evidence that it can also have a positive effect on maternal mental health outcomes, such as postpartum depression, anxiety, stress and distress, and on mother–infant bonding. We found limited evidence that kangaroo mother care has a beneficial effect on father–infant interactions, but no clear effect on paternal depression or stress was observed. Although our review findings are applicable to the mothers and fathers of low-birth-weight and preterm infants globally, including those in low- and middle-income countries, caution is warranted as the certainty of evidence ranges from high to very low. Nevertheless, our findings address an important knowledge gap and could help support the promotion of kangaroo mother care as an intervention that can enhance maternal health in the postnatal period as well as improving the infant’s health. Further research is needed to clarify the effect of kangaroo mother care for vulnerable preterm and low-birth-weight infants on maternal physical health and on paternal health, and to explore the possible biological mechanisms underlying its beneficial effects.
